# Water-Soluble Extract of Pacific Krill Prevents Triglyceride Accumulation in Adipocytes by Suppressing PPARγ and C/EBPα Expression

**DOI:** 10.1371/journal.pone.0021952

**Published:** 2011-07-07

**Authors:** Hidetoshi Yamada, Tomohiro Ueda, Akira Yano

**Affiliations:** 1 Iwate Biotechnology Research Center, Kitakami, Iwate, Japan; 2 Iwate Fisheries Technology Center, Kamaishi, Iwate, Japan; City of Hope National Medical Center and Beckman Research Institute, United States of America

## Abstract

**Background:**

Pacific Krill (*Euphausia pacifica*) are small, red crustaceans, similar to shrimp, that flourish in the North Pacific and are eaten in Japan.

**Methods and Findings:**

We investigated the effect of a water-soluble extract of Pacific Krill on adipocytes and discovered that this extract suppressed triglyceride accumulation in adipocytes. Furthermore, the water-soluble extract of Pacific Krill suppressed the expression of two master regulators of adipocyte differentiation, peroxisome proliferator-activated receptor gamma (PPARγ) and CCAAT enhancer binding protein alpha (C/EBPα). C/EBPβ promotes PPARγ and C/EBPα expression, but the water-soluble extract of Pacific Krill did not inhibit the expression of C/EBPβ or C/EBPβ-mediated transcriptional activation. The Pacific Krill extract was more effective than a PPARγ antagonist in suppressing PPARγ and C/EBPα expression.

**Conclusions:**

These results indicated that the water-soluble extract of Pacific Krill was not simply a PPARγ antagonist, but that it prevented triglyceride accumulation in adipocytes by suppression of PPARγ and C/EBPα via a pathway that is independent of C/EBPβ.

## Introduction

Obesity increases the likelihood of various diseases, including type 2 diabetes and cardiovascular diseases. Foods that suppress absorption of glucose and lipids [1], [2] and increase the metabolism of fat [3]–[6] are regarded as anti-obesity foods. Several anti-obesity foods were studied to identify the bioactive compounds and the physiological function of the compounds that are responsible for their anti-obesity effects. Foods that prevent fat accumulation also seem to be effective in preventing obesity, but only a few studies have investigated the foods that specifically prevent fat accumulation.

Body fat is mainly stored in adipose tissue. Adipose tissue is generally regarded as mesodermal in origin, and mature adipocytes develop from mesenchymal stem cells (MSCs) via a preadipocyte stage [7]. Increases in adipose tissue mass seem to depend on adipocyte hypertrophy because mature adipocytes have low proliferative potential. Recently, however, it was shown that, in adults, the number of adipocytes could increase by adipogenesis, the differentiation of preadipocytes to mature adipocytes [8]. Adipogenesis and adipocyte hypertrophy may occur reiteratively as an individual becomes obese; therefore, inhibition of adipogenesis may be an effective approach to the prevention of fat accumulation.

Adipogenesis is regulated by PPARγ and C/EBPα expression [9]. PPARγ is a nuclear hormone receptor that heterodimerizes with the retinoid X receptor (RXR). The PPARγ-RXR complex promotes the expression of target genes and adipocyte differentiation. C/EBPα is a transcription factor that is required for adipocyte maturation [9].

Pacific Krill are small, red crustaceans, similar to shrimp, that flourish in the North Pacific, and they have been part of Japanese diet for seventy years. They have a high content of long-chain polyunsaturated fatty acids, including eicosapentaenoic acid (EPA) and docosahexaenoic acid (DHA), and several studies report that Krill Oil has anti-inflammatory effects and that it decreases serum lipid levels [10]–[16]. Krill Oil has been studied as a bioactive food, but other constituents of Krill have not been studied intensively. In the present study, we investigated the effect of the water-soluble extract of Pacific Krill on adipocytes and discovered that this extract prevented triglyceride accumulation in adipocytes by suppressing adipogenesis.

## Results

### The water-soluble extract of Pacific Krill suppressed triglyceride accumulation in mouse adipocytes

**Figure 1:**
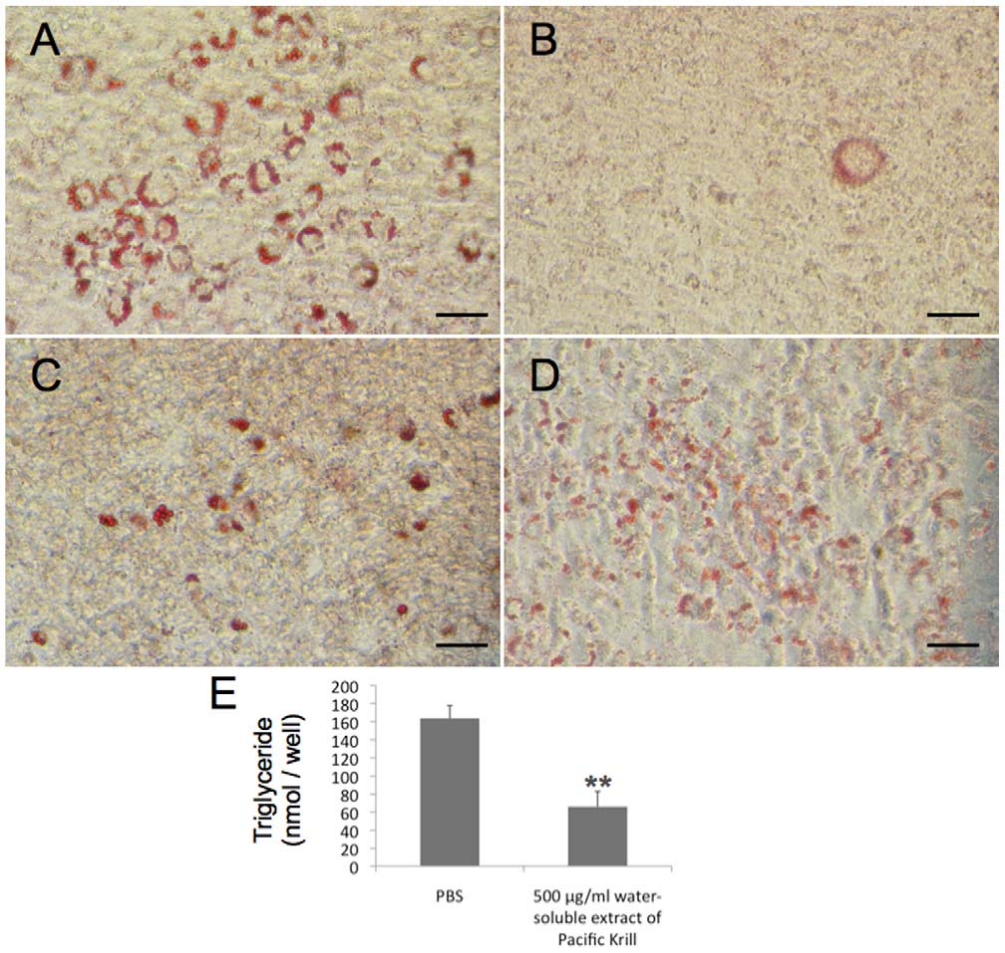
Effect of the water-soluble extract of Pacific Krill on adipocyte differentiation. Oil Red O staining of 3T3-F442A cells that were induced to differentiate as adipocytes and treated with (A) PBS, (B) 500 µg/ml, (C) 50 µg/ml, or (D) 5 µg/ml of the water-soluble extract of Pacific Krill for 10 days. Scale bar, 50 µm. (E) Triglyceride measurements. Plotted values represent the mean value ± SD from ten independent cultures. **p<0.01.

To examine the effect of the water-soluble extract of Pacific Krill on adipocytes, we cultured 3T3-F442A cells in the presence of 10 µg/ml of insulin and 500 µg/ml of the water-soluble extract of Pacific Krill for 10 days. We stained 3T3-F442A cells induced to differentiate as adipocytes with Oil Red O and counted the number of differentiated adipocytes that accumulated triglyceride within the cultures. The water-soluble extract of Pacific Krill suppressed triglyceride accumulation in a dose-dependent manner (Fig. 1). There were few adipocytes that had accumulated triglycerides in the cultures treated with 500 µg/ml of the water-soluble extract (Fig. 1B).

### The water-soluble extract of Pacific Krill did not influence the proliferation or differentiation potential of preadipocytes

**Figure 2 pone-0021952-g002:**
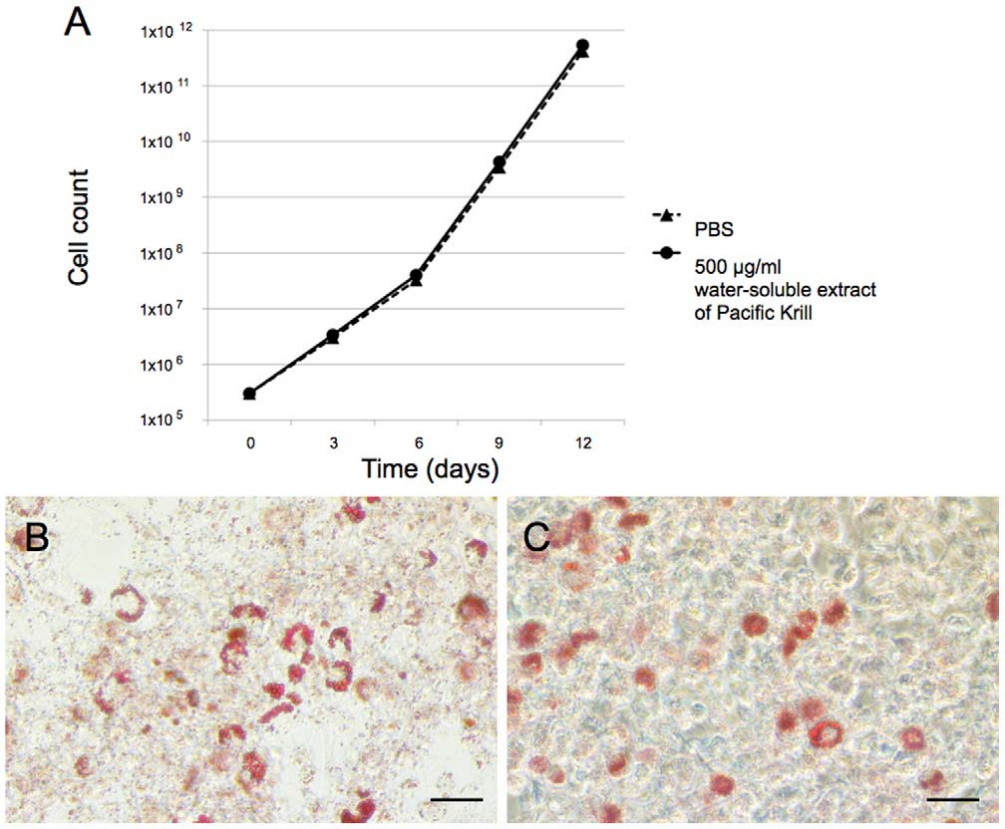
Effects of the water-soluble extract of Pacific Krill on preadipocytes. (A) Growth curve of preadipocytes cultured with insulin-free medium containing PBS (▴) or 500 µg/ml of the water-soluble extract of Pacific Krill (•). The data points represent the mean value ± SD from four independent experiments. (B and C) Oil Red O staining of 3T3-F442A. These cells were cultured in insulin-free medium containing (B) PBS or (C) 500 µg/ml of the water-soluble extract of Pacific Krill for 12 days and then induced to differentiate as adipocytes by adding insulin to the culture medium. Scale bar, 50 µm.

In adipocytes, differentiation and cell cycle progression regulate each other [17]–[19]. To examine the effect of the water-soluble extract of Pacific Krill on preadipocytes proliferation, we cultured 3T3-F442A cells in insulin-free medium and counted the number of cells in the culture every 3 days to assess the influence of the water-soluble extract on preadipocyte proliferation. The water-soluble extract did not inhibit preadipocyte proliferation (Fig. 2A). To determine whether the water-soluble extract affected the differentiation potential of preadipocytes, we cultured 3T3-F442A cells cultured in insulin-free medium containing 500 µg/ml of the water-soluble extract of Pacific Krill for 12 days and then induced adipocyte differentiation by adding insulin (10 µg/ml) to the cultures (Fig. S4B). 3T3-F442A cell cultures pretreated with 500 µg/ml of the water-soluble extract and the control cultures had similar numbers of cell that accumulate triglyceride (Fig. 2B, C).

### The water-soluble extract of Pacific Krill suppressed PPARγ and C/EBPα expression

**Figure 3 pone-0021952-g003:**
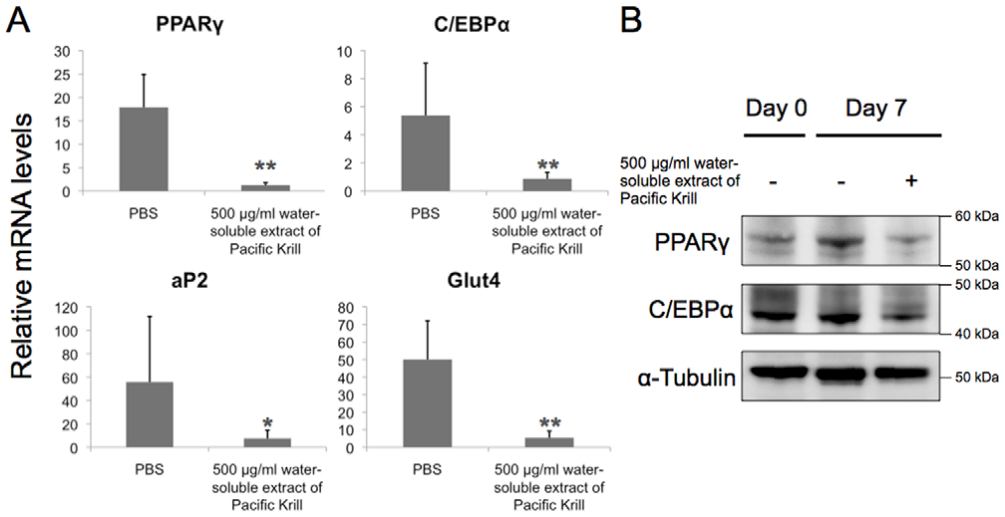
Analysis of PPARγ and C/EBPα mRNA and protein levels in 3T3-F442A cells. (A) qRT-PCR analysis of RNA extracts from 3T3-F442A cells induced to differentiate as adipocytes for 7 days. Plotted values represent the mean value ± SD from five independent cultures. **p<0.01; *p<0.05. (B) Protein extracts from 3T3-F442A cells induced to differentiate as adipocytes for 7 days were analyzed by immunoblotting.

PPARγ and C/EBPα expression are induced during adipogenesis [9]; therefore, we measured PPARγ and C/EBPα expression in 3T3-F442A cells cultured with 10 µg/ml of insulin for 7 days. PPARγ and C/EBPα mRNA expression were induced in the control 3T3-F442A cells, and PPARγ and C/EBPα mRNA levels were significantly lower in the 3T3-F442A cells treated with the water-soluble extract of Pacific Krill than in the control cultures (Figs. 3A, S1). Moreover, PPARγ and C/EBPα protein levels were lower in the extract-treated cultures than in the control cultures (Fig. 3B). The expression of aP2, a gene induced by PPARγ, and Glut4, a marker of mature adipocytes, were also lower in cultures treated with the water-soluble extract Pacific Krill than in control cultures (Fig. 3A). We also examined water-soluble extract of Antarctic Krill (*Euphausia superba*). The water-soluble extract of Antarctic Krill suppressed triglyceride accumulation and PPARγ expression, but the activity was lower than the Pacific Krill extract (Fig. S2).

### The water-soluble extract of Pacific Krill inhibited adipocyte differentiation in human mesenchymal stem cells

**Figure 4:**
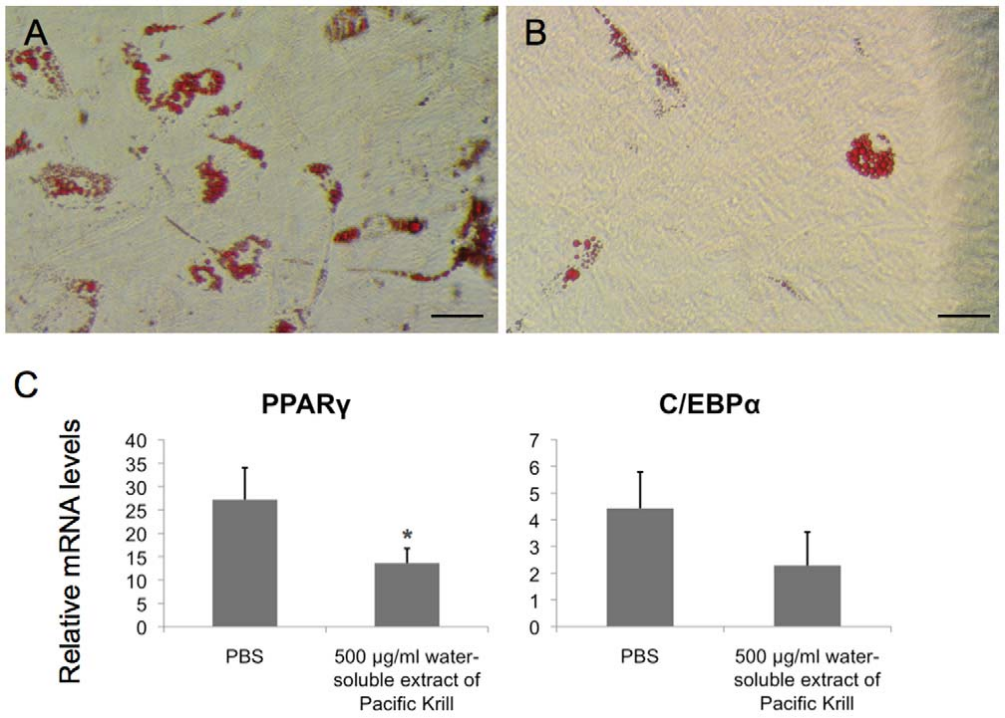
Influence of the water-soluble extract of Pacific Krill on adipocyte differentiation in hMSCs. After 13 days of adipocyte differentiation, (A) PBS or (B) 500 µg/ml of the water-soluble extract of Pacific Krill were added to the cell cultures. (A and B) The Oil Red O staining of UCB TERT-21 cells induced to differentiate as adipocytes for 22 days. Scale bar, 50 µm. (C) RNA extracts from UCB TERT-21 cells induced to differentiate as adipocytes for 18 days were analyzed by qRT-PCR. Plotted values represent the mean value ± SD from four independent cultures. *p<0.05. The p value of C/EBPα was 0.059.

We examined whether the water-soluble extract of Pacific Krill also inhibited adipocyte differentiation of human mesenchymal stem cells (hMSCs). After 13 days of adipocyte differentiation, we added 500 µg/ml of the water-soluble extract of Pacific Krill to hMSC medium and allowed the cultures to grow. We then measured PPARγ and C/EBPα mRNA expression levels in these hMSCs cultures using qRT-PCR, and we stained hMSCs cultures with Oil Red O. The water-soluble extract of Pacific Krill suppressed PPARγ and C/EBPα expression in hMSC cultures (Fig. 4C), and the number of cells that accumulated triglycerides were lower in the extract-treated cultures than in the control cultures (Fig. 4A, B).

### C/EBPβ expression and C/EBPβ-mediated transcriptional activation were not affected by the water-soluble extract of Pacific Krill

**Figure 5:**
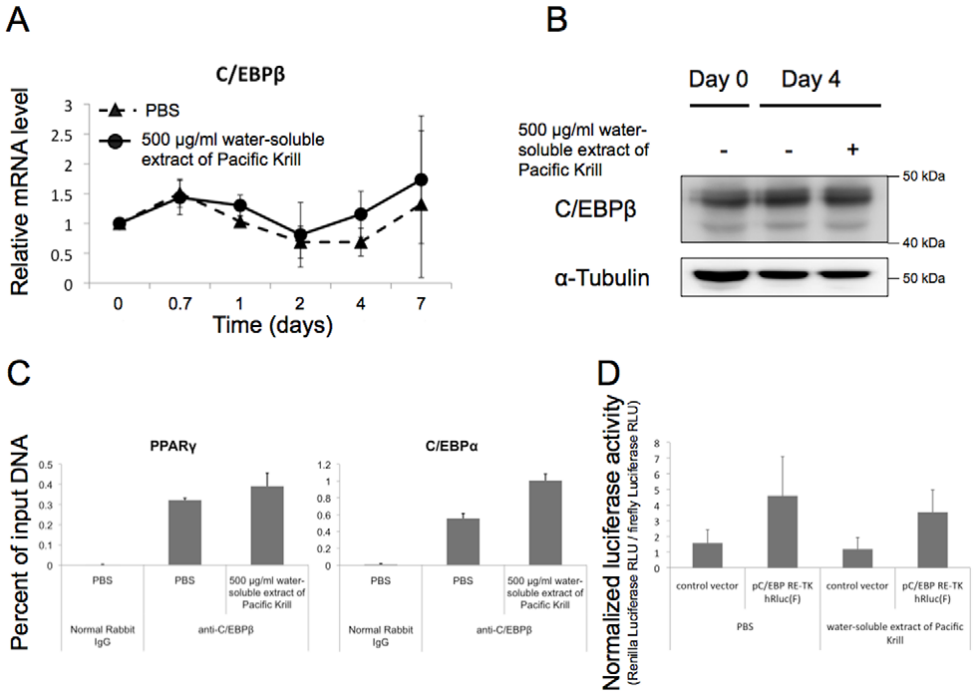
Effects of the water-soluble extract of Pacific Krill on C/EBPβ expression and C/EBPβ-mediated transcriptional activation. (A) qRT-PCR analysis of RNA extracts from 3T3-F442A cells induced adipocyte differentiation. Plotted values are the mean value ± SD from five independent cultures. (B) Protein extracts from 3T3-F442A cells induced to differentiate as adipocyte for 4 days were analyzed by immunoblotting. (C) ChIP analysis of C/EBPβ ChIP samples were extracted from the 3T3-F442A cells induced to differentiate as adipocyte and cultured with or without 500 µg/ml of the water-soluble extract of Pacific Krill for 4 days. Plotted values are the mean value ± SD from three independent experiments. Normal Rabbit IgG was used as a negative control. (D) Luciferase reporter assay. The renilla luciferase activity was normalized to firefly luciferase activity. Plotted values are the mean value ± SD from eight independent experiments.

C/EBPβ binds to the PPARγ and C/EBPα promoters and activates expression of both genes [20]–[22]. We assumed that the water-soluble extract of Pacific Krill decreased the expression of C/EBPβ and C/EBPβ-mediated transcriptional activation. To test these hypotheses, we measured C/EBPβ mRNA expression levels and protein levels in extract-treated and control cultures, but the C/EBPβ mRNA and protein levels were similar in control and extract-treated cultures (Fig. 5A, B). Next, we performed ChIP analysis and luciferase reporter assays to examine C/EBPβ-mediated transcriptional activation. The amount of C/EBPβ bound to the PPARγ and C/EBPα promoters was similar in control and extract-treated cells (Fig. 5C). The reporter activity of pC/EBP RE-TK hRLuc(F) construct was similar in control and extract-treated cultures (Fig. 5D).

### The water-soluble extract of Pacific Krill suppressed PPARγ and C/EBPα expression more than a PPARγ antagonist did

**Figure 6 pone-0021952-g006:**
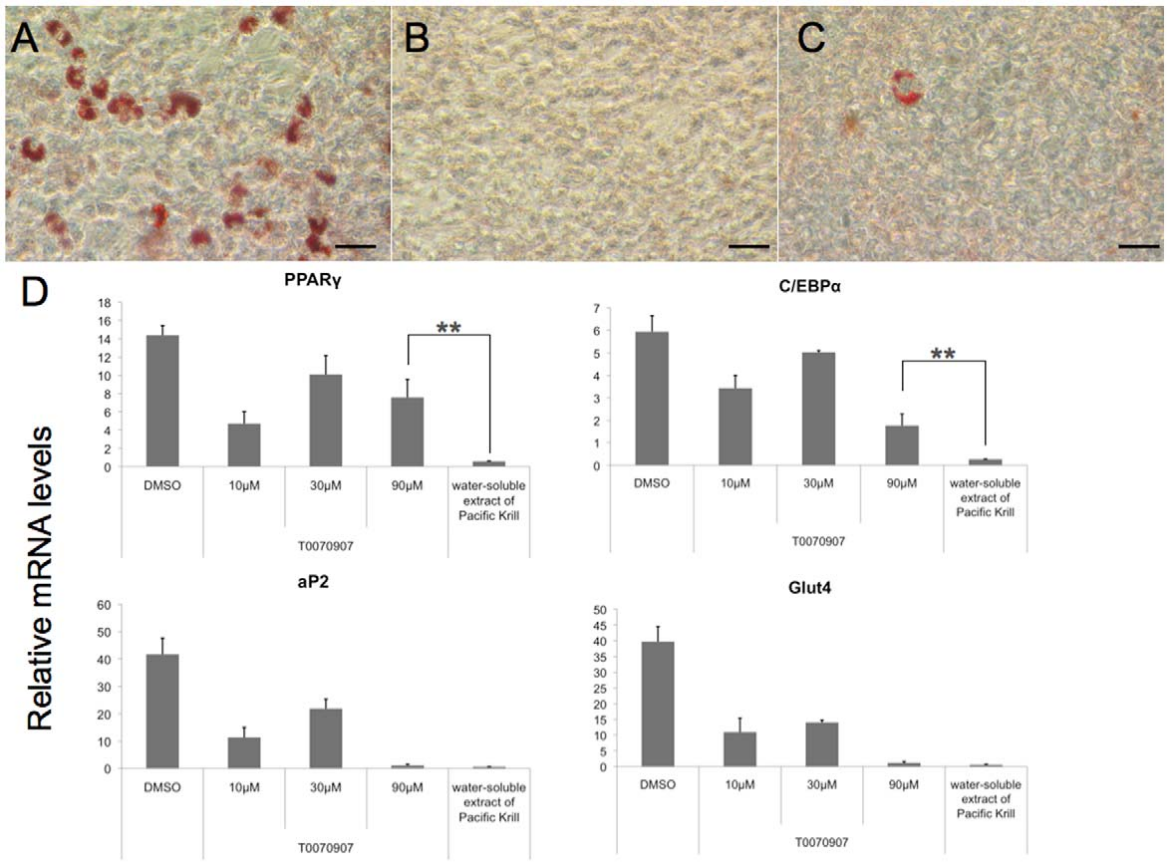
Comparison of effects of the water-soluble extract and a PPARγ antagonist on adipocyte differentiation. Oil Red O staining of 3T3-F442A cells induced to differentiate as adipocytes and treated with (A) DMSO, (B) 500 µg/ml of the water-soluble extract of Pacific Krill, or (C) 10 µM T0070907 for 10 days. Scale bar, 50 µm. (D) Analysis of mRNA levels in 3T3-F442A cells. RNA extracts from 3T3-F442A cells induced to differentiate as adipocytes for 7 days were analyzed by qRT-PCR. Plotted values are the mean value ± SD from four independent cultures. **p<0.01.

The PPARγ antagonists bind to PPARγ selectively and inhibit PPARγ function [23]. Because PPARγ and C/EBPα activate each other, the PPARγ antagonists suppress PPARγ and C/EBPα expression. To assess whether the extract functioned as a PPARγ antagonist, we compared the effects of the water-soluble extract with those of a PPARγ antagonist (T0070907). Triglyceride accumulation was suppressed by 10 µM T0070907 (Fig. 6C), and 90 µM T0070907 suppressed the expression of aP2 to the levels found in cultures treated with the water-soluble extract of Pacific Krill (Fig. 6D). However, while the water-soluble extract of Pacific Krill suppressed more than 95% of PPARγ and C/EBPα expression, T0070907 suppressed less than 70% of PPARγ and C/EBPα expression (Fig. 6D).

### Transcriptome analysis of 3T3-F442A cells treated with the water-soluble extract of Pacific Krill

**Figure 7 pone-0021952-g007:**
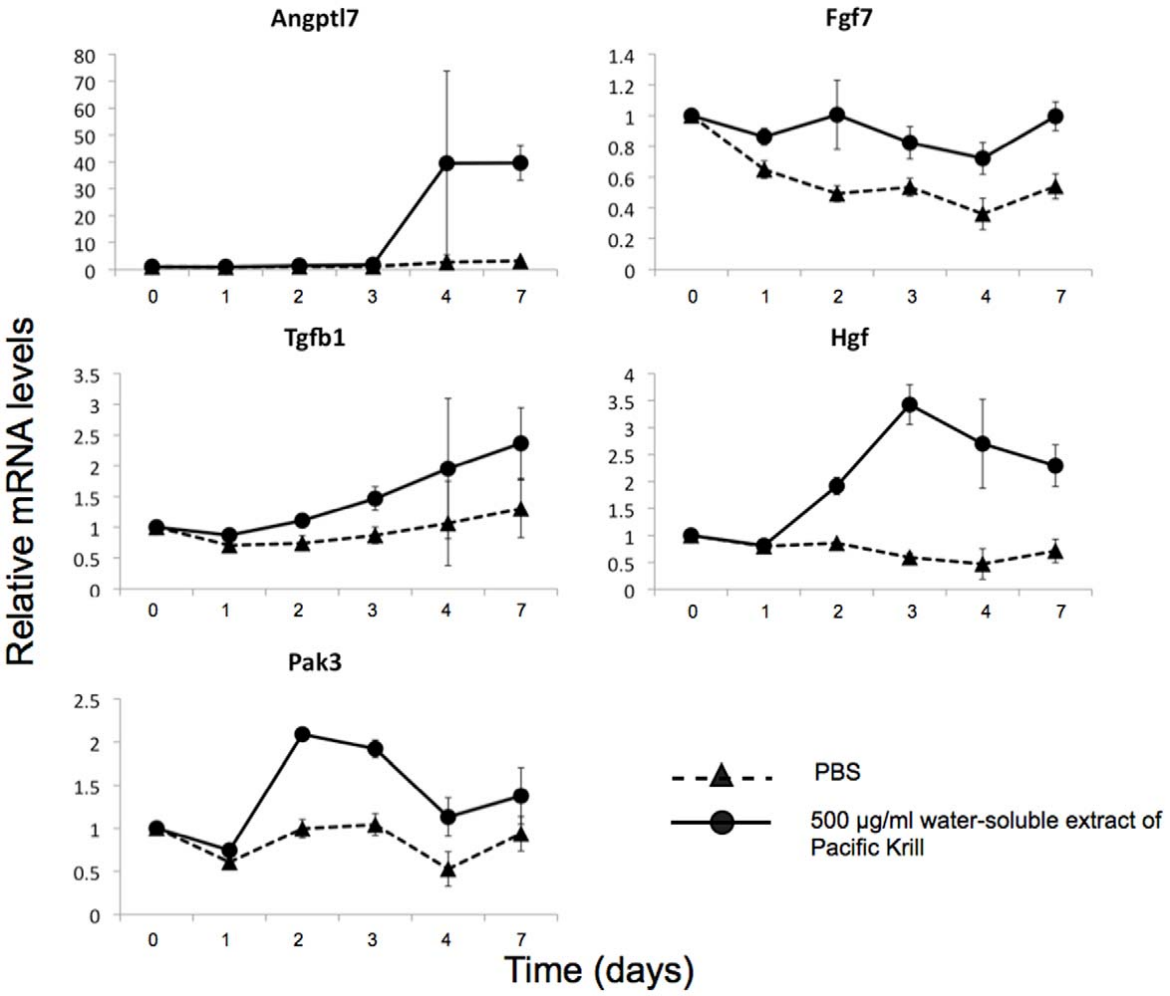
The genes activated by the water-soluble extract of Pacific Krill. qRT-PCR analysis of RNA extracts from 3T3-F442A cells induced to differentiate as adipocytes and treated with PBS (▴) or 500 µg/ml of the water-soluble extract of Pacific Krill (•). Plotted values are the mean value ± SD from four independent cultures.

**Figure 8:**
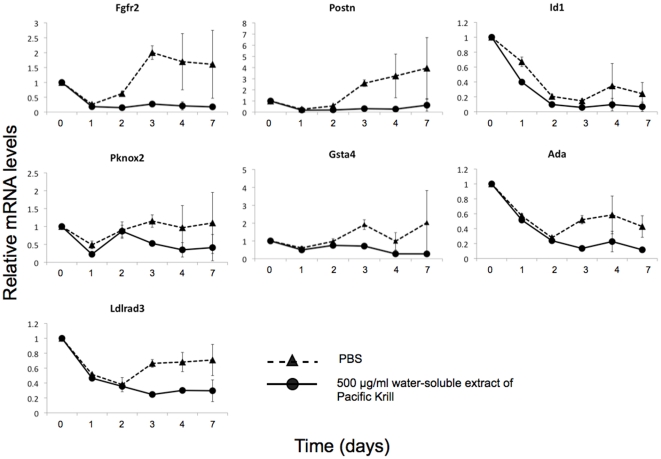
The genes suppressed by the water-soluble extract of Pacific Krill. qRT-PCR analysis of RNA extracts from 3T3-F442A cells induced to differentiate as adipocytes and treated with PBS (▴) or 500 µg/ml of the water-soluble extract of Pacific Krill (•). Plotted values are the mean value ±SD from four independent cultures.

To identify the genes—other than PPARγ and C/EBPα—that were affected by the water-soluble extract of Pacific Krill, we analyzed the transcriptome of 3T3-F442A cells using Super SAGE (serial analysis of gene expression). We identified 65 genes with expression levels that may be affected by treatment with the water-soluble extract by comparing the transcriptome of control 3T3-F442A cells with the transcriptome of 3T3-F442A cells cultured with the water-soluble extract. We used qRT-PCR to confirm that these 65 genes were affected by treatment with the extract. We identified five genes with expression levels that were increased by the water-soluble extract (Fig. 7) and seven genes with expression levels that were decreased (Fig. 8).

## Discussion

We discovered that the water-soluble extract of Pacific Krill prevented triglyceride accumulation by suppressing PPARγ and C/EBPα expression (Figs. 1–4). A study of PPARγ^+/−^ mice shows that appropriate reduction in PPARγ expression prevents obesity induced by a high-fat diet [24]; therefore, inhibition of PPARγ expression may be an effective way to prevent obesity. However, constitutive or complete elimination of PPARγ results in damage to adipose tissue, and this tissue plays a central role in maintaining lipid homeostasis, energy balance, and producing leptin and adiponectin [25]–[29]. Therefore, suppression, rather than elimination of PPARγ expression, may be a better way to prevent obesity. The water-soluble extract of Pacific Krill did not affect proliferation or differentiation of preadipocytes (Fig. 2) and suppressed the expression of PPARγ in adipocytes (Fig. 3). Consumption of the water-soluble extract of Pacific Krill may be a safe and effective way to prevent obesity.

Decreased C/EBPβ expression and DNA binding, inhibition of PPARγ function, and activation of the Wnt/β-catenin pathway suppresses PPARγ and C/EBPα expression [9], [23], [29]–[32]. However, the water-soluble extract of Pacific Krill did not decrease C/EBPβ expression or C/EBPβ-mediated transcriptional activation (Fig. 5), and the extract did not increase the expression of Nr2f2, a gene that acts downstream of Wnt/β-catenin signal to silence PPARγ expression (Fig. S3). Moreover, the extract suppressed PPARγ and C/EBPα expression more than the PPARγ antagonist did (Fig. 6). These results indicate that the water-soluble extract of Pacific Krill was not only a PPARγ antagonist, but that it suppressed the expression of PPARγ and C/EBPα via a pathway other than those mediated by C/EBPβ or Wnt/β-catenin.

The water-soluble extract of Pacific Krill induced expression of five genes—Angptl7, Fgf7, Tgfb1, Hgf, and Pak3; and it suppressed expression of seven genes—Fgfr2, Postn, Id1, Pknox2, Gsta4, Ada, and Ldlrad3. FGF signals positively regulate adipocyte differentiation [33]–[36]. While the expression of Fgf7 was increased approximately two-fold in cells treated with the extract, the expression of Fgfr2 was decreased to approximately one-seventh of the level found in control cells. These findings imply that FGF signaling was blocked in 3T3-F442A cells treated with the extract. TGFβ signal function is antagonistic to PPARγ, and TGFβ induces myoblast differentiation [37]. We propose that decreased FGF signaling and increased TGFβ signaling resulted suppression PPARγ and C/EBPα expression.

Krill Oil has been identified as a bioactive compound, and its functions have been studied, but other components of the krill have not been studied. This study is the first to report that the water-soluble extract of Krill contains bioactive compound. Furthermore, there are only a few reports that indicate that the water-soluble components extracted from a food can suppress the expression of PPARγ; therefore, the bioactive material contained in the water-soluble extract of Krill is of great interest. Previous studies [10]–[13], [38]–[42] and the present results indicate that Krill is beneficial to human health because it has anti-inflammatory effects, decreases serum lipid levels, and prevents fat accumulation in adipocytes.

## Materials and Methods

### Water-soluble extract of Pacific Krill

A mixture of Pacific Krill and distilled water (3∶1 by volume) was homogenized, and the resulting homogenate was centrifuged at 8000 rpm for 30 min. The supernatant was filtered and lyophilized.

### Cell culture

3T3-F442A cells from a mouse preadipocyte cell line (DS pharma Biomedical Co., Ltd., Osaka, Japan) were cultured in DMEM containing 10% Fetal Bovine Serum (FBS) and Antibiotic Antimycotic Solution (Sigma-Aldrich, St Louis, MO, USA). 3T3-F442A cells were grown until confluent on type II collagen-coated dishes (Corning, Corning, NY, USA), and confluent cells were induced to differentiate into adipocytes with 10 µg/ml of insulin (Sigma-Aldrich). The culture conditions used in individual experiments are summarized in Figure S4.

UCB TRET-21 cells, immortalized human Mesenchymal Stem Cells, (JCRB Cell Bank, Osaka, Japan) were cultured in DMEM containing 10% Mesenchymal Stem Cell-qualified FBS (Invitrogen, Carlsbad, CA, USA) and Antibiotic Antimycotic Solution (Sigma-Aldrich). UCB TERT-21 cells were induced to differentiate into adipocytes with 10 µg/ml of insulin, 1 µM dexamethasone (Wako, Osaka, Japan), 200 µM indomethasin (Wako), and 500 µM IBMX, 3-isobutyl-1-methylxanthine (Wako). The culture conditions used in individual experiments are summarized in Figure S4.

### Quantitative real-time PCR (qRT-PCR)

Total RNA was extracted from 3T3-F442A cells or UCB TERT-21 cells with an RNeasy kit (QIAGEN, Tokyo, Japan) and used to synthesize cDNA with the PrimeScript RT reagent kit (Takara, Shiga, Japan); all kits were used according to the manufacturers' recommendations. Quantitative real-time PCR was performed with the gene specific primers listed in Tables S1 and S2 and Fast SYBER Green master mix (Applied Biosystems). The qRT-PCR data from 3T3-F442A cells were normalized to RPLP0. The qRT-PCR data from UCB TERT-21 cells were normalized to Actinβ. The results are expressed as fold increase compared to non-induced 3T3-F442A cells or UCB TRET-21 cells.

### Oil Red O Staining

The cells induced to differentiate as adipocytes were fixed with 10% formalin (Wako) at room temperature for 10 min. Fixed cells were washed with PBS and isopropanol solution and then stained with 180 mg/ml of Oil Red O for 40 min. The Oil Red O-stained cells were observed with a Nikon microscope (×400) after washing. An Adipogenesis Assay kit (Bio Vision, Mountain View, CA, USA) was used to measure triglyceride content.

### Super serial analysis of gene expression (SAGE)

Super SAGE was performed as previously described [43]–[45]. Briefly, total RNA was extracted from 3T3-F442A cells with an RNeasy kit. Double-stranded cDNA was synthesized with biotinated oligo-dT primer and Double-Stranded cDNA Synthesis kit (Invitrogen) from 5 µg of total RNA. Double-stranded cDNAs were digested with NlaIII (New England Biolabs, Beverly, MA, USA) after purification using a PCR purification kit (QIAGEN). Double-stranded cDNAs digested with NlaIII were bound to Dynabeads M270 streptavidin (Invitrogen). Adapter sequence 2 containing an EcoP15I restriction site was ligated to the double-stranded cDNAs that were bound to Dynabeads. Double-stranded cDNAs were digested with EcoP15I (New England Biolabs). Double-stranded cDNAs digested with EcoP15I and separated from Dynabeads were tag sequences. Adaptor sequence 1 containing index sequence was ligated to tag sequences. Tag sequences were then amplified by PCR and gel purified. Finally, tag sequences were analyzed using a Genome Analyzer II (Ilumina, San Diego, CA, USA).

### Super SAGE data analysis

Sorting of sequence reads (35-bases) based on index sequence and the subsequent extraction of 26-base reference sequences from reads was conducted using a script written in C++. The reference sequences were compared to the NCBI mouse transcript database using the BLAST program. We searched the reference sequences, which completely matched the mouse cDNA sequence, Tags. The total number of analyzed reads and tags are shown in Table S3.

### Chromatin immunoprecipitation (ChIP)

ChIP was performed with a ChIP Expression kit (Active motif, Tokyo, Japan). Anti-C/EBPβ (Santa Cruz, Santa Cruz, CA, USA) and normal rabbit IgG (Santa Cruz) were used for ChIP. ChIP samples were analyzed by gene-specific quantitative real-time PCR. The primers used to amplify PPARγ promoter sequences were 5′-CACGCCCCTCACAGAACAGTGAA-3′ and 5′-GCACTGTCCTGACTGAGAGCCA-3′. The primers used to amplify C/EBPα promoter sequences were 5′-ATGCTCCCCACTCACCGCCT-3′ 5′-GCCCCCTGGTGTCCAAACGG-3′. The results are presented as percent of input DNA.

### Plasmids

We used the pC/EBP RE-TK hRluc(F) vector (RIKEN BRC, Ibaraki, Japan), which uses the nucleotide sequence of the C/EBP response element (5′- GATCCGCCAATGCCAATGCCAATG -3′) found upstream of minimal thymidine kinase (TK) promoter in the renilla luciferase reporter construct. The control vector was pmutant C/EBP RE-TK hRluc(R) vector (RIKEN BRC), which contains the mutant C/EBP response element(5′- ACTATGACTATGACTATG -3′) upstream of minimal thymidine kinase (TK) promoter. The luciferase reporter assay normalization vector pCMV-Luc contains the firefly luciferase gene driven by the cytomegarlovirus promoter.

### Luciferase reporter assay

The firefly luciferase normalization vector pCMV-Luc (0.3 µg) and the renilla luciferase reporter construct pC/EBP RE-TK hRluc (F) (3 µg) were co-transfected using Lipofectamin 2000 (Invitrogen) into the 3T3-F442A cells that had been induced to differentiate into adipocytes for 3 days. One day after transfection, the cells were harvested using passive lysis buffer (Promega). Renilla and firefly luciferase activities were determined using a dual luciferase assay system (Promega).

## Supporting Information

Figure S1 **Dose-response curve for the effects of water-soluble extract of Pacific Krill on suppression of PPARγ gene expression.** RNA extracts from 3T3-F442A cells induced to differentiate as adipocytes and treated with the water-soluble extract of Pacific Krill for 7 days were analyzed by qRT-PCR.
(TIFF)

Figure S2 **Effects of the water-soluble extract of Antarctic krill on adipocytes differentiation.** Oil Red O staining of 3T3-F442A cells that were induced to differentiate as adipocytes and treated with (A) PBS or (B) 3 mg/ml of the water-soluble extract of Antarctic Krill for 10 days. Scale bar, 50 µm. (C) qRT-PCR analysis of RNA extracts from 3T3-F442A cells induced to undergo adipocyte differentiation with the water-soluble extract of Antarctic krill for 7 days. Plotted values are the mean value from three independent cultures.
(TIFF)

Figure S3 **Influences of the water-soluble extract of Pacific Krill on Nr2f2 expression.** qRT-PCR analysis of RNA extracts from 3T3-F442A cells induced to undergo adipocyte differentiation for 7 days. Plotted values are the mean value ± SD from three independent cultures.
(TIFF)

Figure S4 **Flow chart of culture conditions (A).** The culture condition of 3T3-F442A cells used to generate the data presented in Figures 1, 3, 5–8. (B) The culture condition of 3T3-F442A cells used to generate the data presented in Figure 2. (C) The culture condition of UCB TERT-21 cells used to generate the data presented in Figure 4.
(TIFF)

Table S1 **The list of primers used for gene expression analysis in 3T3-F442A cells.**
(DOC)

Table S2 **The list of primers used for gene expression analysis in UCB TERT-21 cells.**
(DOC)

Table S3 **Total number of analyzed reads and tags in the Super SAGE analysis.**
(DOC)
