# Supplementary figures and images for: Water-Soluble Extract of Pacific Krill Prevents Triglyceride Accumulation in Adipocytes by Suppressing PPARγ and C/EBPα Expression
Source: PLoS One. 2011 Jul 7;6(7):e21952. doi: 10.1371/journal.pone.0021952 (PMC3131400; doi:10.1371/journal.pone.0021952)

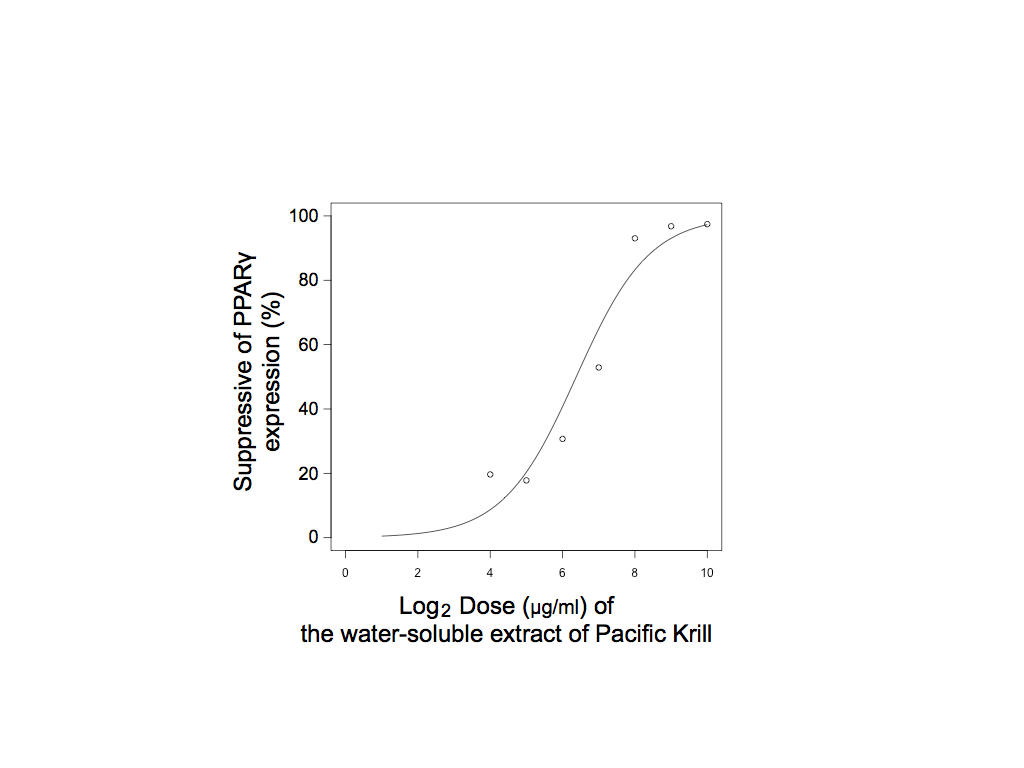

Supplement: Figure S1 — Dose-response curve for the effects of water-soluble extract of Pacific Krill on suppression of PPARγ gene expression. RNA extracts from 3T3-F442A cells induced to differentiate as adipocytes and treated with the water-soluble extract of Pacific Krill for 7 days were analyzed by qRT-PCR. (TIFF) [file pone.0021952.s001.tif]

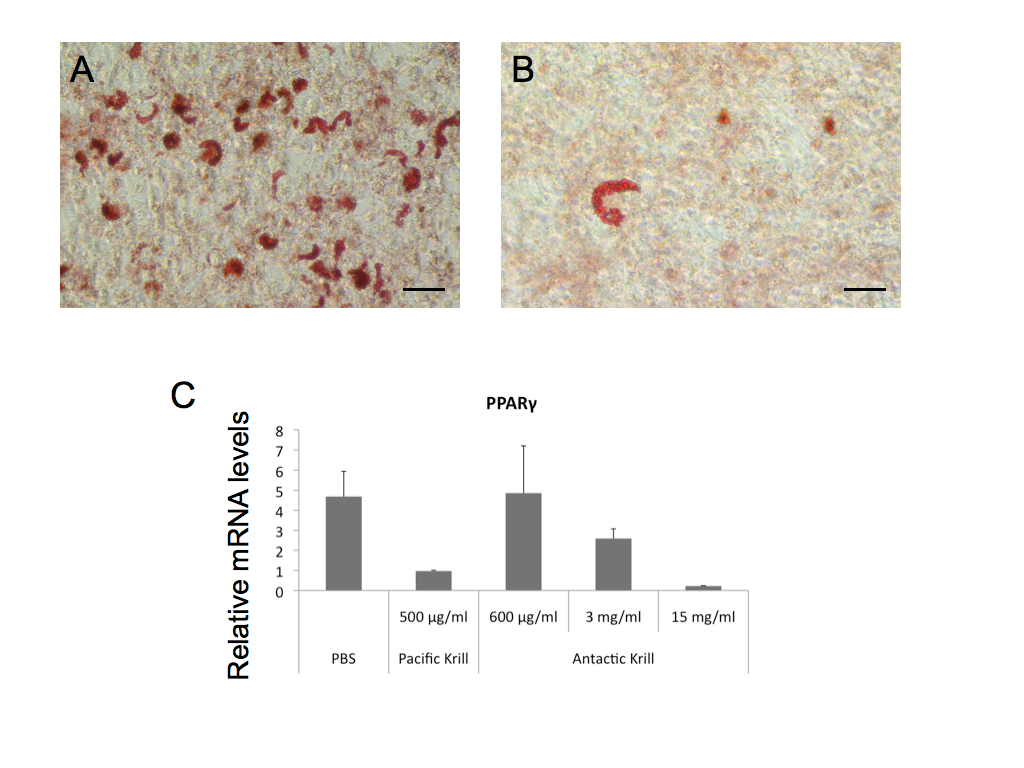

Supplement: Figure S2 — Effects of the water-soluble extract of Antarctic krill on adipocytes differentiation. Oil Red O staining of 3T3-F442A cells that were induced to differentiate as adipocytes and treated with (A) PBS or (B) 3 mg/ml of the water-soluble extract of Antarctic Krill for 10 days. Scale bar, 50 µm. (C) qRT-PCR analysis of RNA extracts from 3T3-F442A cells induced to undergo adipocyte differentiation with the water-soluble extract of Antarctic krill for 7 days. Plotted values are the mean value from three independent cultures. (TIFF) [file pone.0021952.s002.tif]

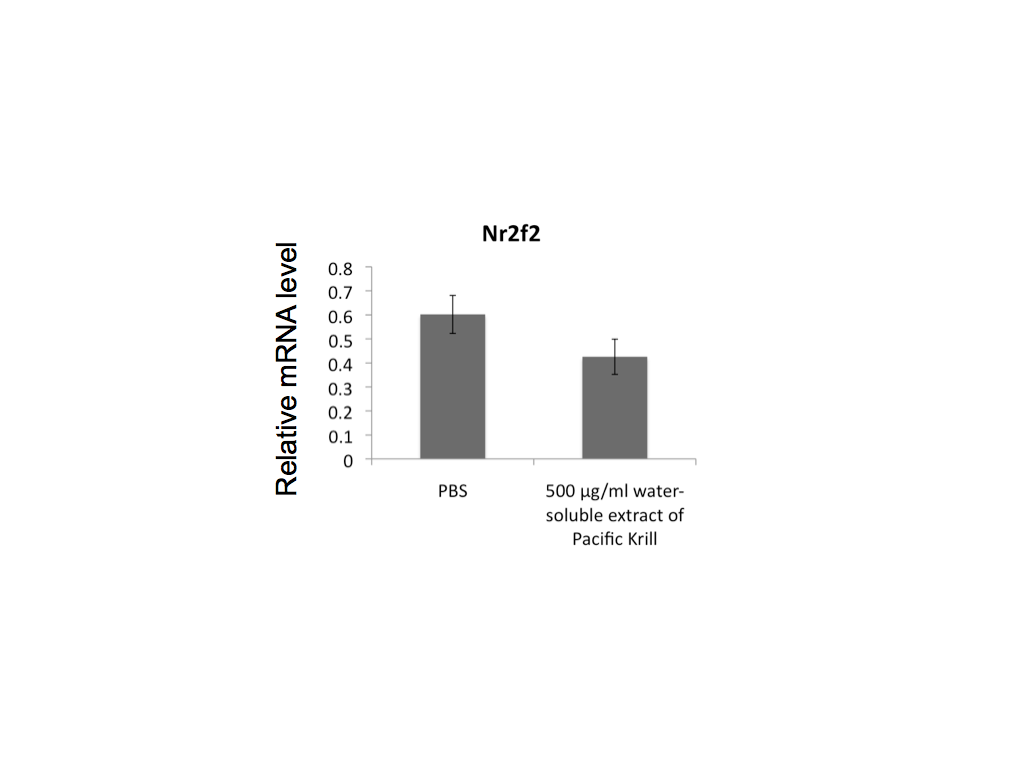

Supplement: Figure S3 — Influences of the water-soluble extract of Pacific Krill on Nr2f2 expression. qRT-PCR analysis of RNA extracts from 3T3-F442A cells induced to undergo adipocyte differentiation for 7 days. Plotted values are the mean value ± SD from three independent cultures. (TIFF) [file pone.0021952.s003.tif]

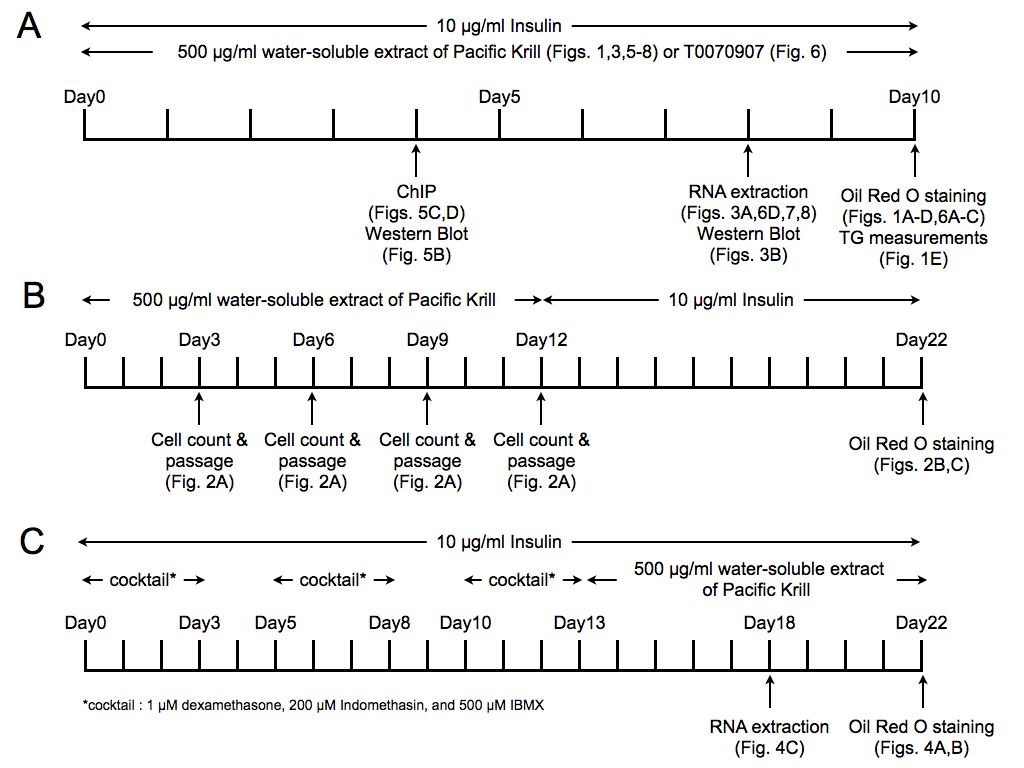

Supplement: Figure S4 — Flow chart of culture conditions (A). The culture condition of 3T3-F442A cells used to generate the data presented in Figures 1, 3, 5– 8. (B) The culture condition of 3T3-F442A cells used to generate the data presented in Figure 2. (C) The culture condition of UCB TERT-21 cells used to generate the data presented in Figure 4. (TIFF) [file pone.0021952.s004.tif]
