# Supplementary material for: Water-Soluble Extract of Pacific Krill Prevents Triglyceride Accumulation in Adipocytes by Suppressing PPARγ and C/EBPα Expression
Source: PLoS One. 2011 Jul 7;6(7):e21952. doi: 10.1371/journal.pone.0021952 (PMC3131400; doi:10.1371/journal.pone.0021952)
Supplement: Table S1 — The list of primers used for gene expression analysis in 3T3-F442A cells. (DOC) [file pone.0021952.s005.doc]

| Mouse primers |  | Sequence |
| --- | --- | --- |
| RPLP0 | Forward | TTTGGGCATCACCACGAAAA |
|  | Reverse | GGACACCCTCCAGAAAGCGA |
| PPARγ | Forward | TGCCTTCGCTGATGCACTGCC |
|  | Reverse | CACGGAGAGGTCCACAGAGCTGA |
| C/EBPα | Forward | CCAACCCCATCCCCAACGGC |
|  | Reverse | GAAGCGGTCCAGCCCTGCTC |
| aP2 | Forward | GATGCCTTTGTGGGAACCT |
|  | Reverse | CTGTCGTCTGCGGTGATTT |
| Glut4 | Forward | GGGGTGGGACAGCCAGCCTA |
|  | Reverse | CCGGCCAGGCCCAACAGATG |
| C/EBPβ | Forward | TCGAACCCGCGGACTGCAAG |
|  | Reverse | CGACGACGACGTGGACAGGC |
