# Supplementary material for: Water-Soluble Extract of Pacific Krill Prevents Triglyceride Accumulation in Adipocytes by Suppressing PPARγ and C/EBPα Expression
Source: PLoS One. 2011 Jul 7;6(7):e21952. doi: 10.1371/journal.pone.0021952 (PMC3131400; doi:10.1371/journal.pone.0021952)
Supplement: Table S2 — The list of primers used for gene expression analysis in UCB TERT-21 cells. (DOC) [file pone.0021952.s006.doc]

| Human primers |  | Sequence |
| --- | --- | --- |
| Actinβ | Forward | ACAGAGCCTCGCCTTTGCCG |
|  | Reverse | ACATGCCGGAGCCGTTGTCG |
| PPARγ | Forward | TGACCCAGAAAGCGATTCCTTCAC |
|  | Reverse | AGTTGGTGGGCCAGAATGGCA |
| C/EBPα | Forward | GGAGTGGTTTGGGGTCGCCG |
|  | Reverse | GCGCAGAGGAAGGGAGGGGA |
