# Supplementary material for: Water-Soluble Extract of Pacific Krill Prevents Triglyceride Accumulation in Adipocytes by Suppressing PPARγ and C/EBPα Expression
Source: PLoS One. 2011 Jul 7;6(7):e21952. doi: 10.1371/journal.pone.0021952 (PMC3131400; doi:10.1371/journal.pone.0021952)
Supplement: Table S3 — Total number of analyzed reads and tags in the Super SAGE analysis. (DOC) [file pone.0021952.s007.doc]

| Sample | Total reads (35-bases) | Total tags (26-bases) |
| --- | --- | --- |
| 3T3-F442A cells treated with Insulin for 4 days. | 753,345 | 532,426 |
| 3T3-F442A cells treated with Insulin and water-soluble extract of Pacific Krill for 4 days. | 916,233 | 678,840 |
